# Antimicrobial resistance dynamics in *Mycobacterium tuberculosis* coinfection systems: A spatiotemporal strain analysis

**DOI:** 10.1016/j.bbrep.2026.102474

**Published:** 2026-02-20

**Authors:** Jie Dai, Fanglin Zeng, Fengyue Hu, Xin Mao, Wenjuan Guo, Weili Liu, Jinfu Wang, Changjiao Luan

**Affiliations:** a Department of Clinical Laboratory, The Third People's Hospital of Yangzhou, Yangzhou, China; b Department of Lung, The Third People's Hospital of Yangzhou, Yangzhou, China; c Laboratory of Intensive Care, Laboratory for Prevention and Translation of Geriatric Diseases, The Affiliated Hospital of Yangzhou University, Yangzhou, China

**Keywords:** Pulmonary tuberculosis, Pulmonary infection, Microbial genus distribution, Bacterial resistance

## Abstract

Secondary pulmonary infections are crucial and pivotal contributing factors to clinical deterioration in patients with pulmonary tuberculosis. Research remains limited regarding the specific population and regional epidemiology of these secondary infections, despite its clinical importance. This study provides an in-depth analysis of the primary bacterial pathogens and their antimicrobial resistance rates among hospitalized pulmonary tuberculosis patients with concurrent pulmonary infections in Yangzhou, China, from 2021 to 2024. Suitable sputum samples and leftover bronchoalveolar lavage fluid samples were collected for bacterial culture identification and drug sensitivity testing. The annual and regional distributions of strains and the evolution of resistance rates were analyzed using the chi-square test and the Cochran-Armitage trend test. A total of 514 strains of pathogenic bacteria were cultured from 410 samples, with 499 strains (97.08 %) being Gram-negative bacteria. *Klebsiella pneumoniae* was the most common Gram-negative bacterium (40.27 %), followed by *Pseudomonas aeruginosa* (15.18 %), while *Staphylococcus aureus* was the primary Gram-positive bacterium (1.75 %). The drug sensitivity tests results over different years showed that *K. pneumoniae* exhibited significant resistance rates against doxycycline, ceftazidime, cefepime, cefuroxime, cefazolin, cefotaxime, levofloxacin, ticarcillin/clavulanate, amikacin, and norfloxacin (P < 0.05). The resistance rates of *P. aeruginosa* to cefazolin and ciprofloxacin were also statistically significant (P < 0.05). The drug sensitivity test results across different regions showed that the resistance rate of *K. pneumoniae* to minocycline in urban patients was statistically significant (P = 0.012), while the resistance rates of *P. aeruginosa* to tobramycin and ciprofloxacin in rural patients were statistically significant (P < 0.05). Annual statistics of extended-spectrum beta-lactamase ESBL-producing *K. pneumoniae* resistant strains showed significant annual increases (P < 0.05). Moreover, data on resistant strains in urban and rural areas indicated that the detection rate of ESBL-producing *Escherichia coli* in rural areas was higher than that in urban areas (P = 0.052), and the detection rate of ESBL-producing *K. pneumoniae* resistant strains in rural areas was significantly higher than in urban areas (P = 0.005). The rise of new carbapenem-resistant *K. pneumoniae* strains in 2023 should be monitored. This study provides strong data support to formulate antibiotic treatment plans for patients with tuberculosis and respiratory infections, reduce the risk of resistant strain transmission, and optimize clinical treatment strategies.

## Highlights

• Novel Focus on Tuberculosis-Pulmonary Co-infection Resistome Dynamics.
• Longitudinal, high-resolution resistance trend analysis.
• Regional-national and urban-rural stratification analysis of antimicrobial resistance.
• Mechanistic insights into ESBL and carbapenem resistance.
• Clinically Actionable Precision Medicine Implications

## 1 Introduction

Tuberculosis remains a major global public health challenge. The World Health Organization's "Global Tuberculosis Report 2025" [1] estimated that approximately 10.7 million new tuberculosis cases will occur worldwide in 2024, with approximately 1.23 million deaths. Tuberculosis remains the leading cause of death from infectious diseases worldwide. Approximately 6.5 % of the global total of new cases occurred in China, with approximately 696,000 patients. China ranks fourth globally in terms of the number of tuberculosis cases, meaning it has one of the world's highest tuberculosis burdens. Therefore, the prevention and control of tuberculosis remain urgent issues.

Patients with tuberculosis often experience lung tissue damage due to *Mycobacterium tuberculosis*, which is frequently accompanied by pulmonary fibrosis, cavitation, and bronchial injury [2]. These structural changes not only impair lung function, but they also significantly weaken local immune defenses. This drastically raises the risk of secondary bacterial infections. Moreover, the systemic immune dysregulation caused by tuberculosis also weakens the body's ability to clear pathogens [3]. When secondary infections occur, they often create a vicious cycle with tuberculosis, exacerbating the inflammatory response, prolonging the course of the disease, and making anti-infection treatment more complex. In areas with lung lesions, due to impaired blood circulation, the penetration of antimicrobial drugs is especially difficult. This makes it easy for bacteria to form biofilms or persistently harbor pathogens, thus promoting the emergence of multidrug-resistant strains [4].

Currently, China has set up several antimicrobial resistance (AMR) monitoring networks, such as the CARSS monitoring network and the CHINET monitoring system, that continuously collect and share resistance data on various pathogens. These data primarily originate from general hospitals and cover a wide range of pathogen and infection types, with no significant direct focus on co-infections in patients with pulmonary tuberculosis. Research regarding the bacterial spectrum and resistance dynamics of pulmonary infections in patients with tuberculosis remains limited, particularly at the regional level. The lack of this type of long-term, specialized resistance monitoring data means that there is no precise guidance for empirical anti-infection treatment for clinical practice, which affects the targeted adjustment of prevention and control strategies. Therefore, research regarding the resistance characteristics of pathogens and annual trends in patients with pulmonary tuberculosis and co-infections is scientifically important.

To fill this research gap, we collected clinical data from patients hospitalized with pulmonary tuberculosis and co-infections at a designated hospital in Yangzhou, China, from 2021 to 2024. The aim of this retrospective analysis was to understand the distribution of major bacterial pathogens, resistance rates, and changes in resistance profiles, and reveal their trends over time. The results provide scientific evidence for the treatment of co-infections in pulmonary tuberculosis, as well as provide insights for the optimization of antimicrobial management and formulation of individualized treatment strategies.

## 2 Materials and methods

### 2.1 General information

This study included 410 patients with pulmonary tuberculosis complicated by pulmonary infections, who were treated at the Third People's Hospital of Yangzhou in Jiangsu Province, China, from January 2021 to December 2024. The majority of tuberculosis patients in Yangzhou area were treated in this hospital. During the study, strains from the same specimen of the same patient were excluded, as well as strains that were contaminated or clinically determined to be colonizing. The inclusion criteria were as follows: (1) patients diagnosed with pulmonary tuberculosis according to the latest domestic and international guidelines for tuberculosis diagnosis and treatment; (2) patients diagnosed with respiratory tract infection according to the relevant criteria of the "Internal Medicine (3rd Edition)" published by the People's Medical Publishing House of China (ISBN: 97871178333). This study was approved by the Medical Research Ethics Committee of Yangzhou, Jiangsu Province (Approval No. YZSYLLPJ-2024-1226-10), and registered with the National Health Security Information Platform of China and the Chinese Medical Research Registration Information System (Registration No. MR-32-25-005768).

### 2.2 Methods

#### 2.2.1 Specimen collection and processing

All respiratory specimens were collected before the patient began antimicrobial therapy. The specimen types included (1) deep sputum collected after morning gargling and (2) bronchoalveolar lavage fluid collected after bronchoscopy. After collection, the specimens were immediately placed in sterile containers and sent to the clinical microbiology lab for processing within 2h.

Laboratory operations were strictly performed in accordance with international standards. The inoculation, culture, and preliminary identification processes referenced the relevant principles in the "Quick Methods for High-Quality Isolate Antimicrobial Susceptibility Testing" document published by the Clinical and Laboratory Standards Institute (CLSI M100-Ed30:2020) [5].

#### 2.2.2 Criteria for qualified specimens

The sputum specimens were subjected to Gram staining and examined under low power microscopy. If the epithelial cells were ≤25 per/LP and white blood cells were ≥10 per/LP, or if the ratio of squamous epithelial cells to white blood cells was <1:2.5, then the specimen was deemed qualified and moved on to the next culture steps.

### 2.3 Bacterial culture, identification, and antimicrobial susceptibility testing

#### 2.3.1 Experimental procedure

The qualified sputum samples and bronchoalveolar lavage fluid specimens were inoculated onto blood agar plates and chocolate agar plates, respectively. The inoculated plates were placed in an incubator kept at a temperature of 35 °C with 5 % CO2 for 18–24 h. After incubation, individual suspicious colonies were selected and identified using matrix-assisted laser desorption/ionization time-of-flight mass spectrometry (MALDI-TOF MS, Autobio ms1000).

Antimicrobial susceptibility testing (AST) was primarily completed using the bioMérieux VITEK 2 automated microbiological analysis system. The interpretation of the AST results followed the standards of the CLSI M100-Ed30:2020 document and the latest breakpoint standards published by the European Committee on Antimicrobial Susceptibility Testing [6].

#### 2.3.2 Quality control strains, reagents, and instruments

The susceptibility testing was performed using the microdilution method, and the results were validated using the following standard quality control strains obtained from the American Type Culture Collection (ATCC): *Escherichia coli* ATCC 25922, *P. aeruginosa* ATCC 27853, and *S. aureus* ATCC 29213 (purchased from Thermo Fisher Scientific). The blood agar plates and chocolate agar plates (without antimicrobial agents) used in the experiments were purchased from Zhengzhou Bosai Technology Co., Ltd. Gram staining solution (rapid method) was obtained from Zhuhai Beisuo Biotechnology Co., Ltd. All of the instruments were calibrated and maintained as recommended by the manufacturers.

### 2.4 Statistical methods

WHONET 2022 software was used to perform strain data filtering and antimicrobial susceptibility testing result analysis. Statistical analysis was performed using SPSS 26.0, and graphs were generated using GraphPad Prism 8. Categorical data are described as frequency (proportion, %). Comparisons between groups were made using the chi-square test or Fisher's exact test, with a two-sided significance level set at α = 0.05. The annual trend analysis for drug-resistant strains was performed using the Cochran-Armitage trend test. Statistical significance was set at P < 0.05, and "-" indicates missing data.

## 3 Results

### 3.1 Characteristics of the pathogen distribution

A total of 410 patients with pulmonary tuberculosis and pulmonary infections were treated between 2021 and 2024. Of these, 77 patients had two bacterial infections, 12 patients had three bacterial infections, and 1 patient had four bacterial infections at different time points (Table S1). A total of 514 pathogen strains were isolated and cultured from these patients. Among them, there were 499 strains of Gram-negative bacteria, with an overall detection rate of 97.08 %. The primary species were the following: *K. pneumoniae* (40.27 %), *P. aeruginosa* (15.18 %), and *Acinetobacter baumannii* (8.95 %), and there were 15 strains of Gram-positive bacteria (2.92 %), primarily *S. aureus* (1.75 %) (Table 1, Fig. 1).

**Fig. 1:**
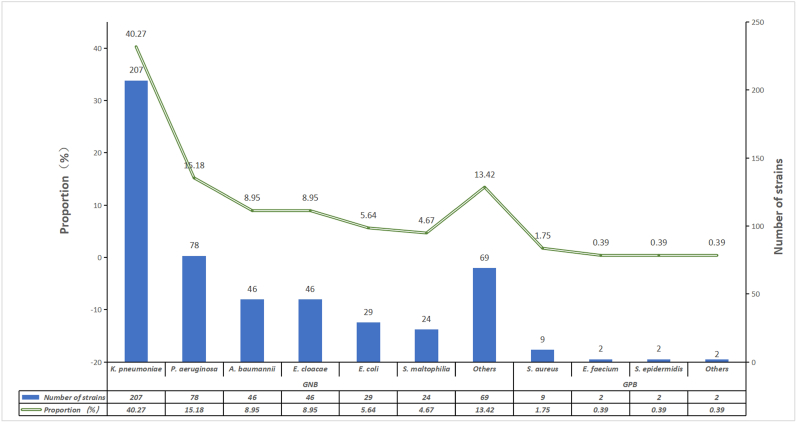
Characteristics of the pathogen distribution Bacterial composition and proportion of secondary bacterial pulmonary infections in pulmonary tuberculosis patients: data refer to table 1.

### 3.2 Specific distribution of gram-negative bacteria by year

The total number of Gram-negative pathogens detected in the hospitalized patients with tuberculosis from 2021 to 2024 was 499 strains, with 61 strains in 2021, 146 strains in 2022, 171 strains in 2023, and 121 strains in 2024. This data indicated a trend that first increased and then decreased. The top five pathogens in each year were consistently *K. pneumoniae*, *P. aeruginosa*, *A. baumannii*, *Enterobacter cloacae*, and *Escherichia coli*, with the order of the bacteria remaining stable. The detection rates of *K. pneumoniae*, *A. baumannii*, and *E. cloacae* peaked in 2023 and showed a decreasing trend in 2024; however, the detection rates of *P. aeruginosa* and *E.* coli increased in 2024 (Table 2).

### 3.3 Specific distribution of gram-negative bacteria by region

The city of Yangzhou includes three urban districts, namely, the Hanjiang District, Guangling District, and Jiangdu District; two county-level cities, namely, Yizheng and Gaoyou; and one county, namely, Baoying County. The top three areas with the most strains detected in patients with tuberculosis and pulmonary infections were: Hanjiang District (175 strains), Guangling District (98 strains), and Jiangdu District (85 strains), which is not surprising given the crowded nature of urban areas and the corresponding higher risk of transmission. The dominant strains detected were primarily *K. pneumoniae*, followed by *P. aeruginosa*, *A. baumannii*, *E. cloacae*, and *E. coli* (Table S2; Fig. S1).

Based on the patients' residential locations and local economic levels, we conducted further analysis of the population sources in urban and rural areas. The results showed that the dominant strain was *K. pneumoniae*, with annual trends generally matching those in urban areas. Notably, the number of strains found in rural areas (*K. pneumoniae*, 110 strains; *P. aeruginosa*, 52 strains; *A. baumannii*, 29 strains; *E. cloacae*, 27 strains; and *E. coli*, 21 strains) was higher than in urban areas (*K. pneumoniae*, 97 strains; *P. aeruginosa*, 26 strains; *A. baumannii*, 17 strains; *E. cloacae*, 19 strains; and *E. coli*, 8 strains). In 2024, the detection rate of *K. pneumoniae* in rural areas decreased compared to the rate in 2023 (from 40.74 % in 2023 to 27.4 % in 2024), while the detection rate in urban areas increased (from 43.66 % in 2023 to 52.94 % in 2024), with *P. aeruginosa* showing the opposite trend (Table S3, Table S4). The annual changes in the key strains showed different epidemiological patterns in urban versus rural areas. Conducting stratified analyzes of urban and rural populations in regional infection monitoring and key population monitoring for tuberculosis is crucial for understanding true epidemiological trends, providing early warnings of potential shifts in risk, and empirically developing clinical medication.

### 3.4 Monitoring of antibiotic resistance rates of gram-negative bacteria by year

Between 2021 and 2024, a total of 207 strains of *K. pneumoniae* were detected, with carbapenem-resistant *K. pneumoniae* (CRKP) first identified in 2023. A total of 71 strains of ESBL-producing *K. pneumoniae* (34.29 %) were detected, showed significant differences. A total of 78 strains of *P. aeruginosa* were detected, including 5 cases (6.41 %) of carbapenem-resistant strains (CRPA). Among the 46 strains of *A. baumannii*, 11 cases (23.91 %) of carbapenem-resistant strains (CRAB) were detected, with detection rates on the rise in 2024. A total of 29 strains of ESBL-producing *E. coli* were detected, of which 25 strains (86.21 %) were ESBL producers (Table 3).

### 3.5 Monitoring of antibiotic resistance rates of gram-negative bacteria in different regions

The distribution in the urban and rural areas of Yangzhou showed that the rural areas had higher numbers and proportions of CRKP, CRPA, CRAB, ESBLs-KP, and ESBLs-Ec strains relative to urban areas. The number of ESBL-producing *K. pneumoniae* was 24 strains in urban areas and 47 strains in rural areas; for ESBL-producing *E. coli*, there were 5 strains in urban areas and 20 strains in rural areas, with chi-square test P-values of 0.005 for ESBLs-KP and 0.052 for ESBLs-Ec strains, respectively. The results indicated that ESBL-resistant strains in patients with tuberculosis and pulmonary infections in rural areas are particularly concerning (Table S5).

### 3.6 Analysis of the antibiotic resistance of *K. pneumoniae*

#### 3.6.1 Analysis of antibiotic resistance of *K. pneumoniae* by year

The resistance spectrum from 2021 to 2024 showed that the five antibiotics with the highest resistance rates in 2021 were moxifloxacin (36.67 %), ciprofloxacin (30.00 %), tetracycline (30.00 %), minocycline (23.33 %), and compound sulfamethoxazole (20.00 %); in 2022, they were moxifloxacin (35.19 %), piperacillin (29.09 %), tetracycline (27.78 %), ciprofloxacin (25.45 %), and cefuroxime and cefotaxime (both 22.22 %); in 2023, they were moxifloxacin (35.62 %), ciprofloxacin (28.00 %), tetracycline (26.03 %), piperacillin (25.33 %), and minocycline (24.00 %); and in 2024, they were moxifloxacin (29.55 %), ciprofloxacin (27.66 %), cefuroxime (27.27 %), cefotaxime (27.27 %), and levofloxacin (25.53 %). The antibiotics with statistically significant differences included doxycycline, ceftazidime, cefepime, cefuroxime, cefazolin, cefotaxime, levofloxacin, ticarcillin/clavulanate, amikacin, and norfloxacin, with the first detection of resistance to doxycycline, cefazolin, and norfloxacin in 2024. Although the number of strains developed resistance to ceftazidime, cefepime, cefuroxime, cefotaxime, levofloxacin, ticarcillin/clavulanate, and amikacin did not change significantly, the proportion of resistant strains increased in 2024, even though the total number of detected resistant strains decreased (Table 4).

#### 3.6.2 Analysis of the antibiotic resistance of *K. pneumoniae* by region

*Klebsiella pneumoniae* was the most common strain found across the different regions of Yangzhou. An analysis of the antibiotic resistance in urban areas showed a statistically significant difference in minocycline resistance (P = 0.012), with newly resistant strains in 2024 that included resistance to doxycycline, cefazolin, ceftazidime, meropenem, imipenem, and norfloxacin (Table S6; Figure S2 A). In rural areas, *K. pneumoniae* showed new resistance to doxycycline, cefazolin, and norfloxacin (Table S7; Figure S2 B).

### 3.7 Analysis of the antibiotic resistance of *P*. *aeruginosa*

#### 3.7.1 Analysis of the antibiotic resistance of *P*. *aeruginosa* by year

In 2021, the four antibiotics with the highest resistance rates were levofloxacin (37.5 %), piperacillin (37.5 %), imipenem (25.00 %), and ceftazidime (25.00 %). In 2022, they were levofloxacin (21.73 %), ticarcillin/clavulanate (17.39 %), and ciprofloxacin (17.39 %). In 2023, they were ticarcillin/clavulanate (20.83 %), piperacillin (16.67 %), and ceftazidime (16.67 %), while in 2024, they were cefoperazone (26.09 %), ticarcillin/clavulanate (21.74 %), piperacillin (13.04 %), and ceftazidime (13.04 %). The antibiotics that had a total resistance rate exceeding 10 % over all four years were piperacillin, ceftazidime, levofloxacin, and ticarcillin/clavulanate, with particular attention to strains resistant to penicillins and quinolones. Ciprofloxacin resistance showed a significant downward trend (p = 0.026), with resistance rates declining to 0 % in both 2023 and 2024. New strains resistant to cefoperazone and cefazoxime emerged in 2024 (Table 5).

#### 3.7.2 Analysis of the antibiotic resistance of *P. aeruginosa* by region

In Yangzhou, the second most dominant strain in the area was *P. aeruginosa*. An analysis of the antibiotic resistance in rural areas revealed statistically significant differences in resistance rates for tobramycin and ciprofloxacin that was due to the absence of resistance in 2023 and 2024 (Table S9 and S10). There were no significant statistical differences in the annual and overall resistance rates of *P. aeruginosa* between urban and rural areas (Table S11; Figure S3 A-C).

### 3.8 Comparative analysis of two major bacterial genera using the 2023 CHINET China antibiotic resistance monitoring data

#### 3.8.1 Comparison of the resistance rates of *K. pneumoniae* using the 2023 CHINET China antibiotic resistance monitoring data

We compared the *K. pneumoniae* detected in the sputum and bronchoalveolar lavage fluid samples from patients with tuberculosis using national monitoring data. The analysis indicated that, except for ciprofloxacin, trimethoprim-sulfamethoxazole, tigecycline, and polymyxin showed no statistical differences, with the remaining P values being <0.05. The resistance rate of *K. pneumoniae* strains at our hospital was lower than the national average (Fig. 2, Table 6).

**Fig. 2:**
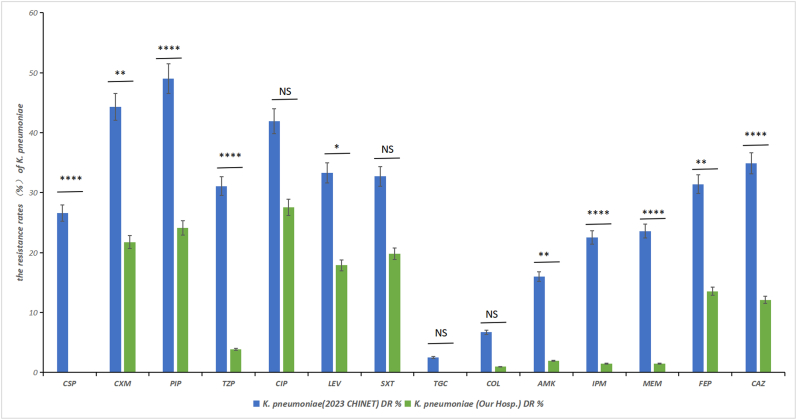
Comparison of *K. pneumoniae* with 2023CHINET Comparison of antibiotic resistance rates: *K*. *pneumoniae* secondary infections in hospitalized pulmonary tuberculosis patients vs. 2023 CHINET data. Data refer to Table 6(∗P < 0.05, ∗∗P < 0.01, ∗∗∗P < 0.001 and ∗∗∗∗P < 0.0001).

#### 3.8.2 Comparison of the resistance rates of *P. aeruginosa* using the 2023 CHINET China antibiotic resistance monitoring data

**Fig. 3:**
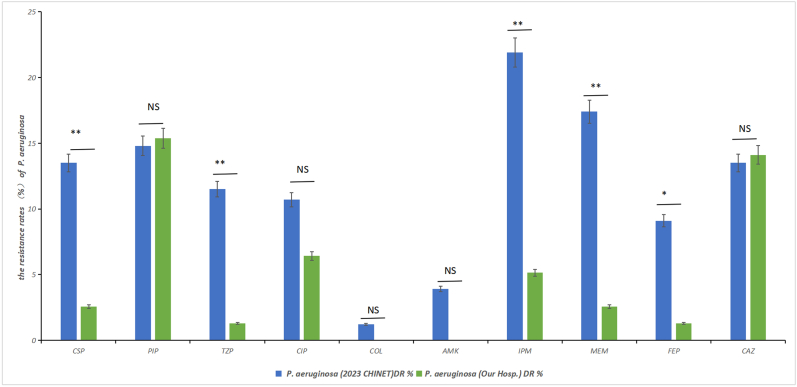
Comparison of *P. aeruginosa* with 2023CHINET Comparison of antibiotic resistance rates: *P. aeruginosa* secondary infections in hospitalized pulmonary tuberculosis patients vs. 2023 CHINET data. Data refer to Table 6(∗P < 0.05, ∗∗P < 0.01, ∗∗∗P < 0.001 and ∗∗∗∗P < 0.0001).

The resistance rate of *P. aeruginosa* strains in patients with tuberculosis was comparable to the national level for most antibiotics (Table 6), with statistically significant antibiotics being cefoperazone/sulbactam (P = 0.009), piperacillin/tazobactam (P = 0.003), imipenem (P = 0.001), meropenem (P = 0.002), and ceftazidime (P = 0.018), all lower than the national level. However, the resistance rates of piperacillin (15.38 %) and ceftazidime (14.1 %) in our hospital were higher than the 2023 CHINET data for piperacillin (14.8 %) and ceftazidime (13.50 %), meriting increased attention and concern (Fig. 3, Table 6).

## 4 Discussion

Pulmonary tuberculosis(TB) is a chronic respiratory infection caused by *Mycobacterium tuberculosis* [7]. Upon pulmonary invasion, *M. tuberculosis* severely affects the respiratory system by impairing bronchial mucosal integrity, causing extensive erosion of lung parenchyma, and disrupting the lung microbiome, increasing the risk for subsequent secondary respiratory infections [2]. Clinically, TB patients with concurrent respiratory infections typically require long-term multidrug regimens [8,9]. However, long-term anti-TB therapy can further perturb airway microbial homeostasis, which can lead to an elevated susceptibility to pathogenic colonization and reinfection; unchecked secondary infections can worsen the severity of the disease and potentially become life-threatening [10,11].

The results of this study showed that Gram-negative bacteria dominated among the pathogens isolated from patients with pulmonary tuberculosis complicated by respiratory infections. This pattern was closely linked to the patients' clinical backgrounds. Structural lung damage from *M.* tuberculosis infection (such as cavity formation and bronchiectasis [12]) often creates a microenvironment conducive to the colonization and proliferation of Gram-negative bacilli [13]. In addition, patients often require repeated or prolonged hospitalization due to their condition, exposing them to the environment of medical institutions, in which they are more likely to encounter hospital-acquired pathogens (usually dominated by Gram-negative bacteria) and a history of prior antibiotic use.

In terms of the specific bacterial distribution, *K. pneumoniae, P. aeruginosa, A. baumannii, E. cloacae*, and *E. coli* were the most frequently isolated bacteria. Antibiotic resistance of *K. pneumoniae* is a continuously evolving global public health issue [14,15]. *Klebsiella* pneumoniae's ability to resist antibiotics stems from two primary pathways: (1) acquired resistance genes, often mobilized by plasmids/transposons, encode β-lactamases and efflux pumps, which can break down antibiotics or pump them out of the bacterial cell, and (2) antibiotic selective pressure leads to adaptive chromosomal mutations and the enhanced ability to form biofilms, which act as a supplementary driver of antibiotic resistance [16,17]. Our 2021–2024 surveillance data align with this evolutionary framework, showing persistently high fluoroquinolone resistance, emergent resistance to doxycycline/cefotaxime/norfloxacin in 2024, elevated β-lactam resistance rates, and the first detection of urban carbapenem resistance in 2024. These shifting antibiotic resistance profiles directly reflect the impact of clinical antibiotic stewardship practices, and these resistance patterns exhibit substantial regional variations. This highlights the necessity for geography-tailored antimicrobial selection and combination therapy to mitigate the development of multidrug resistance [18,19].

*Pseudomonas aeruginosa* is a common pathogen causing severe lung infections [20] by forming protective biofilms that damage the respiratory tract and resist antibiotics and immune attacks [21]. Our 2021–2024 surveillance data show that the drug resistance spectrum of *P*. *aeruginosa* in this region has been continuously evolving. The surveillance data also show that the overall resistance rates of pathogens to piperacillin (a penicillin antibiotic), ceftazidime (a third-generation cephalosporin antibiotic), levofloxacin (a fluoroquinolone antibiotic), and ticarcillin/clavulanic acid (a β-lactam/β-lactamase inhibitor combination) have exceeded 10 %, making them core drugs of concern in clinical practice. *P. aeruginosa* uses efflux pumps, such as MexAB-OprM resistance, as a primary mechanism to pump out β-lactam antibiotics, limiting their effectiveness and contributing substantially to its multidrug resistance [22]. Molecular dynamics simulations have identified nilotinib as a potential inhibitor of the MexB protein [23]. Our surveillance data indicate significant changes in the resistance mechanisms of local strains. The emergence of new resistance to cefotaxime could be a result of the strain acquiring new β-lactamases or upregulating the expression of efflux pumps. Further research into the molecular mechanisms is warranted for confirmation.

Notably, the detection rate of ESBLs-KP has increased over time, while that of ESBLs-EC remains at a high detection level year-round. The detection of resistant strains in rural populations was greater than in urban areas, especially for CRKP, CRPA, CRAB, ESBLs-KP, and ESBLs-EC, where both the number and proportion of detected strains were higher in rural areas than in urban areas. Carbapenem antibiotics (e.g., imipenem, meropenem, and ertapenem) are first-line therapies for infections caused by multidrug-resistant Gram-negative bacteria. However, the rise of carbapenem resistance markedly heightens clinical treatment challenges, leading to high mortality [[24], [25], [26]]. Carbapenem-resistant strains typically harbor both extended-spectrum β-lactamase ESBL genes and carbapenemase-encoding determinants, which further compounds the difficulty of infection control [27,28]. Given that the early symptoms of pulmonary tuberculosis are non-specific, this often leads to the use of antibiotics without specific testing, which may have contributed to the rise of ESBL and CR strains, posing challenges for clinical treatment.

The antibiotic resistance rate of *K. pneumoniae* found in patients with tuberculosis was lower than the national average compared to the 2023 CHINET data. This finding has important clinical implications, suggesting that local patients who require broad-spectrum β-lactam antibiotics might still achieve a high success rate with combinations that include β-lactamase inhibitors (such as piperacillin/tazobactam). It also emphasizes the importance of using carbapenems carefully to ensure they remain effective. For tuberculosis patients with *P. aeruginosa*, it is important to choose drugs such as piperacillin and ceftazidime carefully.

## 5 Conclusion

The results of this study revealed that the predominant pathogens in patients with tuberculosis who have respiratory infections in this region are Gram-negative bacteria, with *K. pneumoniae* and *P. aeruginosa* being the primary strains. We observed changes in how resistant the major pathogens were to specific antimicrobial agents and compared them to the 2023 CHINET data. Notably, temporal shifts in antimicrobial resistance profiles were observed, with β-lactam resistance in *K. pneumoniae* exhibiting an upward trajectory and ciprofloxacin resistance in *P. aeruginosa* showing a progressive decrease over the study period. In our TB cohort, resistance rates were typically lower than national benchmarks, with immediate clinical guidance for empirical antibiotic selection (e.g., potential value of β-lactamase inhibitor combinations.Special attention should be paid to the year-to-year fluctuations in antibiotic resistance among rural patients with tuberculosis complicated by *P*. *aeruginosa* in Yangzhou. These findings provide important insights for empirical anti-infection treatment strategies in this specific population. The characteristics of pathogens in patients with tuberculosis with co-infections change over time and are influenced by multiple factors such as geography, time, and medical practices. The establishment and improvement of a monitoring network for antibiotic resistance in this specific population is of significant scientific value and practical significance for curbing the spread of resistant bacteria, optimizing antimicrobial usage strategies, and improving patient prognoses. Continuous, region-specific epidemiological and resistance monitoring is crucial for guiding timely and effective initial empirical treatment plans for patients with tuberculosis and co-infections.

## Inclusion & ethics

All studies have been approved by the Medical Research Ethics Committee of Yangzhou Third People's Hospital (Approval No.: YZSYLLPJ-2024-1226-10) and have been registered on the National Health Security Information Platform and the Chinese Medical Research Registration and Filing Information System (Registration No.: MR-32-25-005768).

## Limitations and prospects

This study has inherent limitations in analyzing bacterial species distribution and resistance patterns among TB patients with pulmonary infections in Yangzhou, China. First, single-center sampling from Yangzhou Third People's Hospital restricts generalizability to other regions or healthcare settings. Second, distinguishing true respiratory infections from upper respiratory colonization/contamination remains challenging, especially in TB patients with structural lung lesions. Although the 2021–2024 dataset provides valuable resistance data, long-term longitudinal monitoring is needed to develop robust predictive models.

Standardized antibiotic stewardship programs are crucial for controlling the development of resistance to novel antimicrobials in TB patients by promoting the appropriate use of these drugs and ensuring their long-term efficacy. Furthermore, systematic pathogen testing is clinically crucial as it guides personalized therapy and helps reduce selective pressure by enabling the precise identification of the infectious strain and its specific drug resistance profile. This approach, combined with strengthened infection control, helps to curb the transmission of resistant organisms. Multicenter prospective cohort studies are warranted to determine pathogen spectra and track resistance evolution, informing antimicrobial selection for polymicrobial co-infections.

## Author contributions

[Author 1 Jie Dai]: Led the research, conducted experiments, and wrote the manuscript; [Author 2 Fanglin Zeng]: Analyzed data and created figures; [Author 3 Fengyue Hu]: Collected clinical data, recorded data, and participated in statistical analysis; [Author 4 Xin Mao]: Collected clinical samples and participated in sample registration and verification; [Author 5 Wenjuan Guo]: Collected clinical samples and participated in experimental operations; [Corresponding Author 1 Changjiao Luan]: Designed and led the research, revised the manuscript; [Corresponding Author 2 Jinfu Wang]: Guided experimental design, supervised, and reviewed recorded data; [Corresponding Author 3 Weili Liu]: Guided the research and reviewed statistical methods and results.

## Peer review

All authors reviewed and approved the manuscript.

## Declaration of competing interest

The authors declare that they have no known competing financial interests or personal relationships that could have appeared to influence the work reported in this paper

## Figures and Tables

**figs1:**
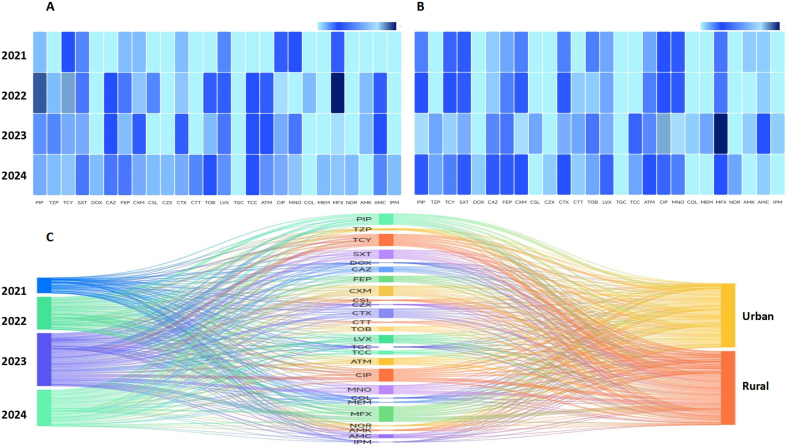


**figs2:**
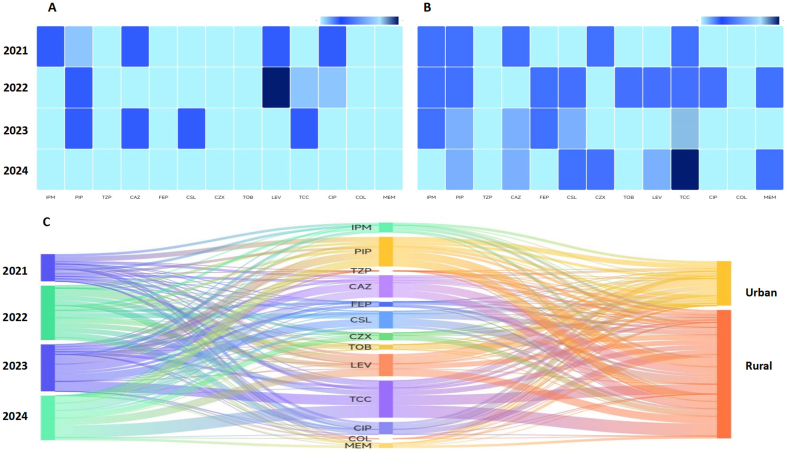


## Data Availability

I have uploaded the raw data involved in the article.
