# Supplementary material for: Antimicrobial resistance dynamics in Mycobacterium tuberculosis coinfection systems: A spatiotemporal strain analysis
Source: Biochem Biophys Rep. 2026 Feb 20;45:102474. doi: 10.1016/j.bbrep.2026.102474 (PMC12937013; doi:10.1016/j.bbrep.2026.102474)
Supplement: Multimedia component 2 [file mmc2.doc]

Table S1. Bacteria Types in Single Secondary Pulmonary Infection with pulmonary tuberculosis

| **Bacteria Types per Patient** | **No. of Patients (cases)** |
| --- | --- |
| 1 Type | 380 |
| 2 Types | 77 |
| 3 Types | 12 |
| 4 Types | 1 |
| Total | 410 |

Table S2. Specific distribution of Gram-negative bacteria in different areas of Yangzhou City

| **Region** | **Hanjiang** | | **Guangling** | | **Jiangdu** | | **Yizheng** | | **Gaoyou** | | **Baoying** | | **Other  regions** | | Total | Annual/Total（%） |
| --- | --- | --- | --- | --- | --- | --- | --- | --- | --- | --- | --- | --- | --- | --- | --- | --- |
| **Annual** | No. of  strains | Proportion(%) | No. of  strains | Proportion(%) | No. of  strains | Proportion(%) | No. of  strains | Proportion(%) | No. of  strains | Proportion(%) | No. of  strains | Proportion(%) | No. of  strains | Proportion(%) |
| 2021 | 23 | 37.70 | 9 | 14.75 | 7 | 11.48 | 4 | 6.56 | 7 | 11.48 | 10 | 16.39 | 1 | 1.64 | 61 | 11.87 |
| 2022 | 46 | 30.67 | 35 | 23.34 | 29 | 19.33 | 18 | 12.00 | 11 | 7.33 | 11 | 7.33 | 0 | 0 | 150 | 29.18 |
| 2023 | 68 | 37.98 | 30 | 16.77 | 28 | 15.64 | 25 | 13.97 | 14 | 7.82 | 7 | 3.91 | 7 | 3.91 | 179 | 34.82 |
| 2024 | 38 | 30.65 | 24 | 19.35 | 21 | 16.94 | 15 | 12.10 | 12 | 9.68 | 9 | 7.26 | 5 | 4.02 | 124 | 24.13 |
| **Region /Total** | 175 | 34.05 | 98 | 19.07 | 85 | 16.54 | 62 | 12.06 | 44 | 8.56 | 37 | 7.20 | 13 | 2.52 | 514 | 100 |


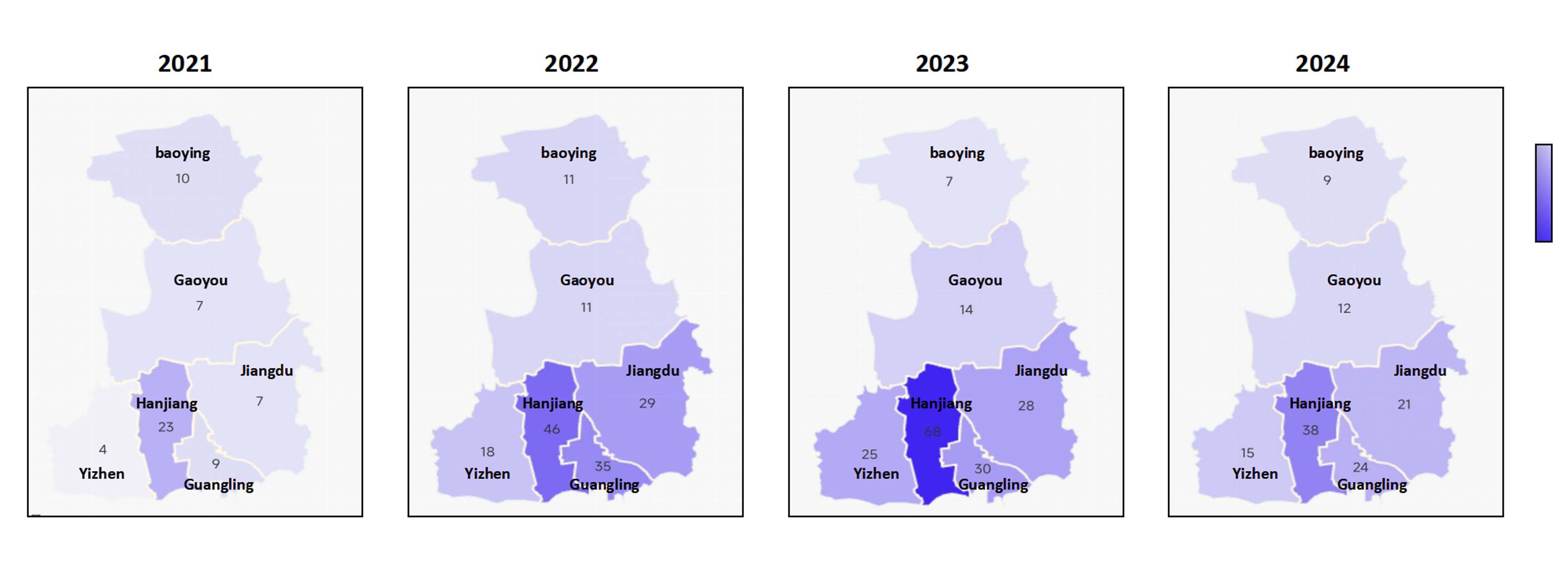
**Figure S1. Specific distribution of Gram-negative bacteria in different areas of Yangzhou City**

Table S3. Specific distribution of Gram-negative bacteria in urban

| **Pathogenic  bacteria** | **2021** | | **2022** | | **2023** | | **2024** | | **Grand Total** | |
| --- | --- | --- | --- | --- | --- | --- | --- | --- | --- | --- |
|  | **No. of  strains** | **Proportion(%)** | **No. of  strains** | **Proportion(%)** | **No. of  strains** | **Proportion(%)** | **No. of  strains** | **Proportion(%)** | **Total No.  of strains** | **total proportion(%)** |
| K. pneumoniae | 13 | 52.00% | 26 | 38.80% | 31 | 43.66% | 27 | 52.94% | 97 | 45.33% |
| P. aeruginosa | 3 | 12.00% | 14 | 20.90% | 7 | 9.86% | 2 | 3.92% | 26 | 12.15% |
| A. baumannii | 3 | 12.00% | 4 | 5.97% | 7 | 9.86% | 3 | 5.88% | 17 | 7.94% |
| E. cloacae | 0 | 0.00% | 9 | 13.43% | 8 | 11.27% | 2 | 3.92% | 19 | 8.88% |
| E. coli | 0 | 0.00% | 1 | 1.49% | 3 | 4.23% | 4 | 7.84% | 8 | 3.74% |
| S. maltophilia | 3 | 12.00% | 4 | 5.97% | 4 | 5.63% | 4 | 7.84% | 15 | 7.01% |
| K. oxytoca | 1 | 4.00% | 0 | 0.00% | 0 | 0.00% | 1 | 1.96% | 2 | 0.93% |
| S. marcescens | 0 | 0.00% | 0 | 0.00% | 3 | 4.23% | 0 | 0.00% | 3 | 1.40% |
| E. aerogenes | 0 | 0.00% | 2 | 2.99% | 2 | 2.80% | 0 | 0.00% | 4 | 1.87% |
| Other GNB | 2 | 8.00% | 3 | 4.48% | 3 | 4.23% | 6 | 11.76% | 14 | 6.54% |
| GPB | 0 | 0.00% | 4 | 5.97% | 3 | 4.23% | 2 | 3.94% | 9 | 4.21% |
| Total No. of strains | 25 | 100.00 | 67 | 100.00 | 71 | 100.00 | 51 | 100.00 | 214 | 100.00 |

Table S4. Specific distribution of Gram-negative bacteria in rural

| **Pathogenic  bacteria** | **2021** | | **2022** | | **2023** | | **2024** | | **Grand Total** | |
| --- | --- | --- | --- | --- | --- | --- | --- | --- | --- | --- |
|  | **No. of  strains** | **Proportion(%)** | **No. of  strains** | **Proportion(%)** | **No. of  strains** | **Proportion(%)** | **No. of  strains** | **Proportion(%)** | **Total No.  of  strains** | **total proportion(%)** |
| K. pneumoniae | 17 | 47.22% | 29 | 34.94% | 44 | 40.74% | 20 | 27.40% | 110 | 36.67% |
| P. aeruginosa | 5 | 13.89% | 9 | 10.84% | 17 | 15.74% | 21 | 28.77% | 52 | 17.33% |
| A. baumannii | 2 | 5.56% | 11 | 13.25% | 10 | 9.26% | 6 | 8.22% | 29 | 9.66% |
| E. cloacae | 4 | 11.11% | 10 | 12.05% | 9 | 8.33% | 4 | 5.48% | 27 | 9.00% |
| E. coli | 5 | 13.89% | 3 | 3.61% | 6 | 5.56% | 7 | 9.59% | 21 | 7.00% |
| S. maltophilia | 0 | 0.00% | 5 | 6.02% | 2 | 1.85% | 2 | 2.74% | 9 | 3.00% |
| K. oxytoca | 0 | 0.00% | 3 | 3.61% | 1 | 0.93% | 1 | 1.37% | 5 | 1.67% |
| S. marcescens | 0 | 0.00% | 1 | 1.23% | 3 | 2.78% | 2 | 2.74% | 6 | 2.00% |
| E. aerogenes | 1 | 2.77% | 3 | 3.61% | 0 | 0.00% | 1 | 1.37% | 5 | 1.67% |
| Other GNB | 2 | 5.56% | 9 | 10.84% | 13 | 12.03% | 6 | 8.22% | 30 | 10.00% |
| GPB | 0 | 0.00% | 0 | 0.00% | 3 | 2.78% | 3 | 4.10% | 6 | 2.00% |
| Total No.  of strains | 36 | 100.00 | 83 | 100.00 | 108 | 100.00 | 73 | 100.00 | 300 | 100.00 |

Table S5. Monitoring of antibiotic resistance rates of Gram-negative bacteria in different regions

| **MDROs** |  | **Urban** |  | **Rural** |  | **Grand  Total** |  | ***χ2*** | ***P值*** |
| --- | --- | --- | --- | --- | --- | --- | --- | --- | --- |
|  |  | **No. of strains** | **Proportion(%)** | **No. of strains** | **Proportion(%)** | **Total No.  of strains** | **total proportion**  **(%)** |  |  |
| CRKP （Total n=207,Urban n=97,Rural n=110) | Total | 97 |  | 110 |  | 207 |  |  |  |
|  | DR | 1 | 1.03 | 3 | 2.73 | 4 | 1.93 | *0.783* | >0.05 |
| CRPA   (Total n=78,Urban n=26,Rural n=52) | Total | 26 |  | 52 |  | 78 |  |  |  |
|  | DR | 1 | 3.85 | 4 | 7.69 | 5 | 6.41 | 0.427 | >0.05 |
| CRAB   (Total n=46,Urban n=17,Rural n=29) | Total | 17 |  | 29 |  | 46 |  |  |  |
|  | DR | 3 | 17.6 | 8 | 27.6 | 11 | 23.91 | 0.582 | >0.05 |
| ESBLs-KP （Total n=207，Urban n=97,Rural n=110) | Total | 97 |  | 110 |  | 207 |  |  |  |
|  | DR | 24 | 24.7 | 47 | 42.7 | 71 | 34.30 | 7.399 | 0.005 |
| ESBLs-EC (Total n=29，Urban n=8,Rural n=21) | Total | 8 |  | 21 |  | 29 |  |  |  |
|  | DR | 5 | 62.5 | 20 | 95.24 | 25 | 86.21 | 5.222 | 0.052 |

**Table S6. Analysis of the antibiotic resistance of *K. pneumoniae*** in urban by year

|  |  |  |  |  |  |  |  |
| --- | --- | --- | --- | --- | --- | --- | --- |
| **Antibacterial Agents** | **2021** | **2022** | **2023** | **2024** | **Grand Total** | ***χ2*** | ***P value*** |
|  | **（n＝13）** | **（n＝26）** | **（n＝31）** | **（n＝27）** | **（n=97）** |  |  |
| PIP | 1（7.69） | 11（42.31） | 6（19.35） | 7（25.93） | 25（25.77） | 6.152 | ＞0.05 |
| TZP | 0（0.00） | 1（3.85） | 2（6.45） | 1（3.70） | 4（4.12） | 0.924 | ＞0.05 |
| TCY | 3（23.08） | 10（0.00） | 7（22.58） | 8（29.63） | 28（28.87） | 1.932 | ＞0.05 |
| SXT | 2（15.38） | 5（19.23） | 7（22.58） | 5（18.52） | 19（19.59） | 0.374 | ＞0.05 |
| DOX | 0（0.00） | 0（0.00） | 0（0.00） | 1（3.70） | 1（1.03） | 2.821 | ＞0.05 |
| CAZ | 0（0.00） | 3（11.54） | 3（9.68） | 5（18.52） | 11（11.34） | 2.716 | ＞0.05 |
| FEP | 1（7.69） | 5（19.23） | 1（3.23） | 5（18.52） | 12（12.37） | 4.791 | ＞0.05 |
| CXM | 1（7.69） | 8（30.77） | 4（12.90） | 7（25.93） | 20（20.62） | 4.285 | ＞0.05 |
| CSL | 0（0.00） | 2（7.69） | 0（0.00） | 1（3.70） | 3（3.09） | 2.674 | ＞0.05 |
| CZX | 0（0.00） | 0（0.00） | 0（0.00） | 1（3.70） | 1（1.03） | 2.821 | ＞0.05 |
| CTX | 1（7.69） | 8（30.77） | 4（12.90） | 7（25.93） | 20（20.62） | 4.285 | ＞0.05 |
| CTT | 0（0.00） | 0（0.00） | 0（0.00） | 2（7.41） | 2（2.06） | 3.386 | ＞0.05 |
| TOB | 0（0.00） | 4（15.38） | 1（3.23） | 3（11.11） | 8（8.25） | 3.591 | ＞0.05 |
| LVX | 2（15.38） | 4（15.38） | 5（16.13） | 6（22.22） | 17（17.53） | 0.642 | ＞0.05 |
| TGC | 0（0.00） | 0（0.00） | 0（0.00） | 0（0.00） | 0（0.00） | - | ＞0.05 |
| TCC | 0（0.00） | 3（11.54） | 3（9.68） | 3（11.11） | 9（9.28） | 1.399 | ＞0.05 |
| ATM | 0（0.00） | 4（15.38） | 3（9.68） | 6（22.22） | 13（13.40） | 3.883 | ＞0.05 |
| CIP | 4（30.77） | 9（34.62） | 7（22.58） | 7（25.93） | 27（27.84） | 1.226 | ＞0.05 |
| MNO | 3（23.08） | 0（0.00） | 5（16.13） | 8（29.63） | 16（16.49） | **10.261** | **0.012** |
| COL | 0（0.00） | 1（3.85） | 0（0.00） | 0（0.00） | 1（1.03） | 2.897 | ＞0.05 |
| MEM | 0（0.00） | 0（0.00） | 0（0.00） | 1（3.70） | 1（1.03） | 2.821 | ＞0.05 |
| MFX | 4（30.77） | 12（46.15） | 9（29.03） | 8（29.63） | 33（34.02） | 2.278 | ＞0.05 |
| NOR | 0（0.00） | 0（0.00） | 0（0.00） | 1（3.70） | 1（1.03） | 2.821 | ＞0.05 |
| AMK | 0（0.00） | 1（3.85） | 1（3.23） | 0（0.00） | 2（2.06） | 1.723 | ＞0.05 |
| AMC | 0（0.00） | 4（15.38） | 3（9.68） | 2（7.41） | 9（9.28） | 2.141 | ＞0.05 |
| IPM | 0（0.00） | 0（0.00） | 0（0.00） | 1（3.70） | 1（1.03） | 2.821 | ＞0.05 |

**Table S7. Analysis of the antibiotic resistance of *K. pneumoniae*** in rural by year

|  |  |  |  |  |  |  |  |
| --- | --- | --- | --- | --- | --- | --- | --- |
| **Antibacterial Agents** | **2021** | **2022** | **2023** | **2024** | **Grand Total** | ***χ2*** | ***P value*** |
|  | **（n＝17）** | **（n＝29）** | **（n＝44）** | **（n＝20）** | **n=110** |  |  |
| PIP | 3（17.65） | 5（17.24） | 13（29.55） | 4（20.00） | 25（22.73） | 1.799 | ＞0.05 |
| TZP | 0（0.00） | 0（0.00） | 2（4.55） | 2（10.00） | 4（3.64） | 3.199 | ＞0.05 |
| TCY | 6（35.29） | 5（17.24） | 12（27.27） | 3（15.00） | 26（23.64） | 2.978 | ＞0.05 |
| SXT | 4（23.53） | 4（13.79） | 10（22.73） | 4（20.00） | 22（20.00） | 1.143 | ＞0.05 |
| DOX | 0（0.00） | 0（0.00） | 0（0.00） | 1（5.00） | 1（0.91） | 3.745 | ＞0.05 |
| CAZ | 1（5.88） | 1（3.45） | 8（18.18） | 4（20.00） | 14（12.73） | 4.945 | ＞0.05 |
| FEP | 2（11.76） | 3（10.34） | 7（15.91） | 4（20.00） | 16（14.55） | 1.114 | ＞0.05 |
| CXM | 3（17.65） | 4（13.79） | 13（29.55） | 5（25.00） | 25（22.73） | 2.691 | ＞0.05 |
| CSL | 0（0.00） | 0（0.00） | 2（4.55） | 0（0.00） | 2（1.82） | 1.976 | ＞0.05 |
| CZX | 0（0.00） | 0（0.00） | 0（0.00） | 1（5.00） | 1（0.91） | 3.745 | ＞0.05 |
| CTX | 3（17.65） | 4（13.79） | 10（22.73） | 5（25.00） | 22（20.00） | 1.322 | ＞0.05 |
| CTT | 0（0.00） | 1（3.45） | 2（4.55） | 1（5.00） | 4（3.64） | 0.967 | ＞0.05 |
| TOB | 3（17.65） | 1（3.45） | 7（15.91） | 2（10.00） | 13（11.82） | 3.427 | ＞0.05 |
| LVX | 2（11.76） | 2（6.90） | 10（22.73） | 6（30.00） | 20（18.18） | 5.359 | ＞0.05 |
| TGC | 0（0.00） | 0（0.00） | 0（0.00） | 0（0.00） | 0（0.00） | - | - |
| TCC | 0（0.00） | 0（0.00） | 6（13.64） | 2（10.00） | 8（7.27） | 5.74 | ＞0.05 |
| ATM | 2（11.76） | 3（10.34） | 8（18.18） | 5（25.00） | 18（16.36） | 2.141 | ＞0.05 |
| CIP | 5（29.41） | 5（17.24） | 14（31.82） | 6（30.00） | 30（27.27） | 2.146 | ＞0.05 |
| MNO | 4（23.53） | 4（13.79） | 13（29.55） | 3（15.00） | 24（21.82） | 3.022 | ＞0.05 |
| COL | 0（0.00） | 0（0.00） | 1（2.27） | 0（0.00） | 1（0.91） | 2.168 | ＞0.05 |
| MEM | 0（0.00） | 0（0.00） | 2（4.55） | 0（0.00） | 2（1.82） | 1.976 | ＞0.05 |
| MFX | 7（41.18） | 7（24.14） | 17（38.64） | 5（25.00） | 36（32.73） | 2.727 | ＞0.05 |
| NOR | 0（0.00） | 0（0.00） | 0（0.00） | 2（10.00） | 2（1.82） | 5.187 | ＞0.05 |
| AMK | 1（5.88） | 0（0.00） | 1（2.27） | 0（0.00） | 2（1.82） | 2.446 | ＞0.05 |
| AMC | 1（5.88） | 1（3.45） | 5（11.36） | 1（5.00） | 8（7.27） | 1.585 | ＞0.05 |
| IPM | 0（0.00） | 0（0.00） | 1（2.27） | 0（0.00） | 1（0.91） | 2.168 | ＞0.05 |

**Table S8. Comparison of the antibiotic resistance of** between urban and rural

| **Antibacterial  Agents** | **Urban  2021-2024** | **Rural  2021-2024** | ***χ2*** | ***P value*** |
| --- | --- | --- | --- | --- |
|  | **（n＝97）** | **（n＝110）** |  |  |
| PIP | 25(25.77) | 25(22.73) | 0.261 | ＞0.05 |
| TZP | 4(4.12) | 4(3.64) | 0.033 | ＞0.05 |
| TET | 28(28.87) | 26(23.64) | 0.731 | ＞0.05 |
| SXT | 19(19.59) | 22(20.00) | 0.006 | ＞0.05 |
| DOX | 1(1.03) | 1(0.91) | 0.008 | ＞0.05 |
| CAZ | 11(11.34) | 14(12.73) | 0.093 | ＞0.05 |
| FEP | 12(12.37) | 16(14.55) | 0.208 | ＞0.05 |
| CXM | 20(20.62) | 25(22.73) | 0.135 | ＞0.05 |
| CSL | 3(3.09) | 2(1.82) | 0.355 | ＞0.05 |
| CZX | 1(1.03) | 1(0.91) | 0.008 | ＞0.05 |
| CTX | 2(2.06) | 2(1.82) | 0.016 | ＞0.05 |
| CTT | 20（0.00） | 22(20.00) | 0.012 | ＞0.05 |
| TOB | 2(2.06) | 4(3.64) | 0.454 | ＞0.05 |
| LEV | 8(8.25) | 13(11.82) | 0.721 | ＞0.05 |
| TGC | 17(17.53) | 20（0.00） | 0.015 | ＞0.05 |
| TCC | 9(9.28) | 8(7.27) | 0.275 | ＞0.05 |
| ATM | 13(13.40) | 18(16.36) | 0.355 | ＞0.05 |
| CIP | 27(27.84) | 30（0.00） | 0.008 | ＞0.05 |
| MIN | 16(16.49) | 24(21.82) | 0.937 | ＞0.05 |
| CLS | 1(1.03) | 1(0.91) | 0.008 | ＞0.05 |
| MEM | 1(1.03) | 2(1.82) | 0.224 | ＞0.05 |
| MXF | 33(34.02) | 36(32.73) | 0.039 | ＞0.05 |
| NOR | 1(1.03) | 2(1.82) | 0.224 | ＞0.05 |
| AMK | 2(2.06) | 2(1.82) | 0.061 | ＞0.05 |
| AMC | 9(9.28) | 8(7.27) | 0.275 | ＞0.05 |
| IPM | 1(1.03) | 1(0.91) | 0.008 | ＞0.05 |

**Table S9. Analysis of the antibiotic resistance of *P. aeruginosa*** in urban by year

| **Antibacterial Agents** | **2021** | **2022** | **2023** | **2024** | **Grand Total** | ***χ2*** | ***P value*** |
| --- | --- | --- | --- | --- | --- | --- | --- |
|  | **（n＝3 ）** | **（n＝14）** | **（n＝ 7 ）** | **（n＝2）** | **n= 26** |  |  |
| IPM | 1（33.33） | 0（0.00） | 0（0.00） | 0（0.00） | 1（3.85） | 5.579 | ＞0.05 |
| PIP | 2（66.67） | 1（7.14） | 1（14.29） | 0（0.00） | 4（15.38） | 5.342 | ＞0.05 |
| TZP | 0（0.00） | 0（0.00） | 0（0.00） | 0（0.00） | 0（0.00） | - | - |
| CAZ | 1（33.33） | 0（0.00） | 1（14.29） | 0（0.00） | 2（7.69） | 4.781 | ＞0.05 |
| FEP | 0（0.00） | 0（0.00） | 0（0.00） | 0（0.00） | 0（0.00） | - | - |
| CSL | 0（0.00） | 0（0.00） | 1（14.29） | 0（0.00） | 1（3.85） | 3.884 | ＞0.05 |
| CZX | 0（0.00） | 0（0.00） | 0（0.00） | 0（0.00） | 0（0.00） | - | - |
| TOB | 0（0.00） | 0（0.00） | 0（0.00） | 0（0.00） | 0（0.00） | - | - |
| LEV | 1（33.33） | 3（21.43） | 0（0.00） | 0（0.00） | 4（15.38） | 2.781 | ＞0.05 |
| TCC | 0（0.00） | 2（14.29） | 1（14.29） | 0（0.00） | 3（11.54） | 1.027 | ＞0.05 |
| CIP | 1（33.33） | 2（14.29） | 0（0.00） | 0（0.00） | 3（11.54） | 2.722 | ＞0.05 |
| COL | 0（0.00） | 0（0.00） | 0（0.00） | 0（0.00） | 0（0.00） | - | - |
| MEM | 0（0.00） | 0（0.00） | 0（0.00） | 0（0.00） | 0（0.00） | - | - |

**Table S10. Analysis of the antibiotic resistance of *P. aeruginosa*** in rural by year

| **Antibacterial Agents** | **2021** | **2022** | **2023** | **2024** | **Grand Total** | ***χ2*** | ***P value*** |
| --- | --- | --- | --- | --- | --- | --- | --- |
|  | **（n＝5 ）** | **（n＝9 ）** | **（n＝ 17 ）** | **（n＝21）** | **n= 52** |  |  |
| IPM | 1（20.00） | 1（11.11） | 1（5.88） | 0（0.00） | 3（5.77） | 4.216 | ＞0.05 |
| PIP | 1（20.00） | 1（11.11） | 3（17.65） | 3（14.29） | 8（15.38） | 0.704 | ＞0.05 |
| TZP | 0（0.00） | 0（0.00） | 0（0.00） | 0（0.00） | 0（0.00） | - | - |
| CAZ | 1（20.00） | 0（0.00） | 3（17.65） | 3（14.29） | 7（13.46） | 1.978 | ＞0.05 |
| FEP | 0（0.00） | 1（11.11） | 1（5.88） | 0（0.00） | 2（3.85） | 2.964 | ＞0.05 |
| CSL | 0（0.00） | 2（22.22） | 3（17.65） | 1（4.76） | 6（11.54） | 2.957 | ＞0.05 |
| CZX | 1（20.00） | 0（0.00） | 0（0.00） | 2（9.52） | 3（5.77） | 3.583 | ＞0.05 |
| TOB | 0（0.00） | 2（22.22） | 0（0.00） | 0（0.00） | 2（3.85） | **5.858** | **0.035** |
| LEV | 0（0.00） | 2（22.22） | 0（0.00） | 3（14.29） | 5（9.62） | 4.07 | ＞0.05 |
| TCC | 1（20.00） | 2（22.22） | 4（23.53） | 5（23.81） | 12（23.08） | 0.268 | ＞0.05 |
| CIP | 0（0.00） | 2（22.22） | 0（0.00） | 0（0.00） | 2（3.85） | **5.858** | **0.035** |
| COL | 0（0.00） | 0（0.00） | 0（0.00） | 0（0.00） | 0（0.00） | - | - |
| MEM | 0（0.00） | 1（11.11） | 0（0.00） | 1（4.76） | 2（3.85） | 2.542 | ＞0.05 |

**Table S11. Comparison of the antibiotic resistance of *P. aeruginosa*** between urban and rural

| **Antibacterial  Agents** | **Urban  2021-2024** | **Rural  2021-2024** | ***χ2*** | ***P value*** |
| --- | --- | --- | --- | --- |
|  | **（n＝26）** | **（n＝52）** |  |  |
| IPM | 1（3.85） | 3（5.77） | 0.132 | ＞0.05 |
| PIP | 4（15.38） | 8（15.38） | 0.000 | ＞0.05 |
| TZP | 0（0.00） | 0（0.00） | - | - |
| CAZ | 2（7.69） | 7（13.46） | 0.565 | ＞0.05 |
| FEP | 0（0.00） | 2（3.85） | 1.026 | ＞0.05 |
| CSP | 1（3.85） | 6（11.54） | 1.256 | ＞0.05 |
| CZX | 0（0.00） | 3（5.77） | 1.560 | ＞0.05 |
| TOB | 0（0.00） | 2（3.85） | 1.026 | ＞0.05 |
| LEV | 4（15.38） | 5（9.62） | 0.656 | ＞0.05 |
| TCC | 3（11.54） | 12（23.08） | 1.486 | ＞0.05 |
| CIP | 3（11.54） | 2（3.85） | 1.710 | ＞0.05 |
| COL | 0（0.00） | 0（0.00） | - | - |
| MEM | 0（0.00） | 2（3.85） | 1.026 | ＞0.05 |
